# Work-family balance and the subjective well-being of rural women in Sichuan, China

**DOI:** 10.1186/s12905-019-0871-6

**Published:** 2020-01-02

**Authors:** Yue Shui, Dingde Xu, Yi Liu, Shaoquan Liu

**Affiliations:** 1 Institute of Mountain Hazards and Environment, Chinese Academy of Sciences, #9, Block 4, Renminnan Road, Chengdu, 610041 China; 2 University of Chinese Academy of Sciences, #19, Yuquan Road, Shijingshan District, Beijing, 100049 China; 3 Sichuan Center for Rural Development Research, College of Management of Sichuan Agricultural University, Chengdu, 611130 China; 4 College of resources, Sichuan Agricultural University, # 211, Huimin Road, Wenjiag District, Chengdu, 611130 China

**Keywords:** Work-family conflict, Subjective well-being, Rural women, China

## Abstract

**Background:**

Due to the great burden of family and the conflicts among family, society and career roles resulting from migrant working, rural women suffer more conflicts between work and family and need more social attention. Previous studies of the conflicts between family and work mainly focus on the group of career women, and there is a lack of the research on the conflicts between work and family of rural women, which needs to be systematically and further studied.

**Methods:**

This study used a sample survey of 380 rural women in rural areas of Sichuan Province to measure rural women’s cognition of work-family, coordination and handling of conflicts, post-conflict choices, and subjective well-being; the study constructs an ordered multi-class logistic regression model to explore the impact of work-family conflict on the subjective well-being of rural women in rural regions.

**Results:**

The study result shows that: (1) The level of subjective well-being of rural women is generally high, and 70% of women feel satisfied or very satisfied. (2) The factor which impacts the subjective well-being among rural women most is work-interfering-with-family conflict, followed by work-family balance and confidence in conflict coordination.

**Conclusion:**

This study can enhance our understanding of rural women in rural areas, and provide a reference for formulating policies to improve people’s life satisfaction.

## Introduction

Urbanization and rural labor migration are the inevitable social development trend accompanied by regional industrialization and economic modernization. Western economics uses “urbanization” to summarize the formation and development of a city and its role in the process of industrialization in a society, considering that the prominent feature of this process is the migration of rural population to cities [1]. Urban areas include cities and towns. Since the reform and opening up, China’s urbanization has developed rapidly, and rural laborers have migrated to urban areas on a large scale. By 2017, the total number of migrant workers in China had reached 280 million [2].

With the deepening of non-agriculturalization, the proportion of women in migrant workers has been increasing. In the past 30 years, the proportion of women in migrant laborers has increased from about 25 to 37% [3], “Breadwinning men and homemaking women” and “farming men and weaving women”, the traditional social patterns of the gender division of labor, have begun to change, and women’s social roles have shifted from the single role of family to the dual roles of career and family [4].

The society has double expectations for women. Specifically, on the one hand, women are expected to actively participate in the social work and contribute to the society; on the other hand, women are expected to play a good family role [5]. Traditional Chinese thought and ethics believe that women should take on more family responsibilities in the family, such as taking care of parents and children, doing housework, etc., and when numerous women go out to work, the time and energy of participating in family activities and fulfilling family responsibilities will be greatly limited [6]. Due to the great burden of family population and the conflicts among family, society and career roles resulting from migrant working, rural women suffer more conflicts between work and family and need social attention. Previous studies of the conflicts between family and work mainly focus on the group of career women, and there is a lack of the research on the conflicts between work and family of rural women, which needs to be systematically and further studied.

Studies have shown that women are always more stressed than men, and in particular, the full-time women whose children are less than 13 years old are the most stressed, while the most common stresses are mainly from family, work and economy [7]. Women’s pursuit of work achievements and their own social values is contrary to traditional moral concepts. Many rural women are unable to go out to work as they wish because of taking care of their families, leading to the deviation between the employment expectations and the actual employment selections. This makes women often suffer from mental struggles and disturbances and results in the decrease in women’s personal well-being. Existing studies have highlighted the correlation between the conflicts between family and work and the subjective well-being. In recent years, domestic and foreign scholars have also conducted the research on the driving factors of subjective well-being from economic status, social conditions, livelihood strategies and other aspects [8, 9]. However, in addition to focusing on the correlation between the conflicts between family and work and the subjective well-being of rural women, this study systematically brings family burden and employment situation into the driving factors of subjective well-being.

Sichuan is a large agricultural province, which has 50% of the rural population [10]. For the rural areas where human capital is scarce, the most practical and effective way to increase income is to go out to work, and due to the problem of going out to work, women in rural areas may have a stronger sense of conflicts [11, 12]. Firstly, based on this, this study takes the rural areas of Sichuan Province, the typical rural province in Western China, as the research area, taking rural women as the research object. Meanwhile, it uses the sample survey data of 400 rural women to analyze the basic characteristics of the conflicts between family and work and the subjective well-being of rural women. Furthermore, it constructs the econometric models to explore the correlation between them, so as to enhance our understanding of the relationship between them and then pertinently provide reference for the formulation of relevant policies. This study intends to explore the following key scientific issues:

1. What are the characteristics of the conflicts between work and family of rural women?
2. How do the conflicts between work and family affect the subjective well-being of rural women?

## Literature review

**Fig. 1:**
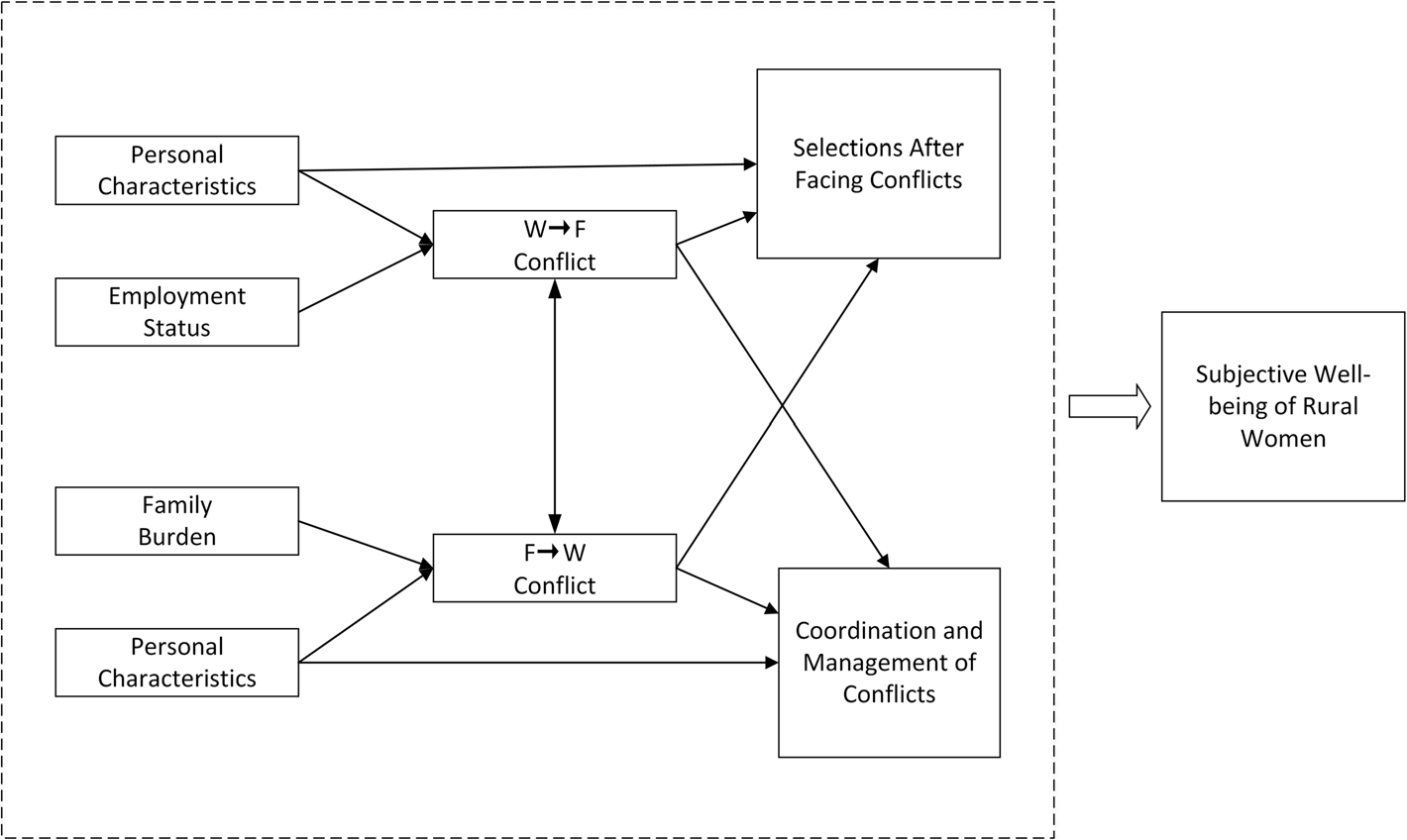
The model of the conflicts between work and family

Early studies were mostly centered on “role” and pointed out that the conflicts between work and family were the main source of role conflicts, rarely focusing on the conflicts between family and work. The model centered on role conflict proposed by Koplman believed that work conflict and family conflict affected role conflict [13], which affected work and family satisfaction and ultimately affected life satisfaction. In 1991, Higgins and Duxbury added two variables, namely role involvement and role expectation [14], putting forward a more complete model. Frone and Cooper extended the previous studies and subdivided the conflicts between work-interfering-with-family (WIF) conflict and family-interfering-with-work (FIW) conflict [15], proposing the bi-directional model of the conflicts between work and family. Their study found that there was a positive correlation between the two forms of conflict, and that job stressors, job involvement, household involvement, and family stressors all had significant effects on FIW and WIF. Referring to the above research, this study proposes the following framework for rural women in China (Fig. 1).

So far, Western scholars have carried out quite a lot of empirical research on the conflicts between work and family, and have obtained abundant research results [16]. Compared with Western countries, China’s studies of the conflicts between work and family are still in the infancy and are mainly reflected in the short time of research and the narrow scope of research, and the measurement tools used in most studies are translation or revision of foreign questionnaires. Due to China’s urban-rural dual structure, there are remarkable differences in the employment market environment between foreign countries and China, and Chinese women’s conflicts between work and family and the influencing factors are also different from foreign women’s. Therefore, this study will take into account the actual situation of rural female laborers in China to construct a new scale, so as to better measure the conflicts between work and family of rural women in China.

Gender is the social demographical characteristic of the conflicts between work and family, which has been most extensively studied by scholars. On the one hand, some studies have argued that women have suffered more negative effects of family-work conflict, and men have suffered more negative influences of work-family conflict; on the other hand, some studies have suggested that compared with men, women have suffered more negative impacts of work (rather than family) [17]. Many Chinese scholars have found that the role conflicts of women participating in employment exist objectively, and women’s extensive participation in employment leads to more conflicts between family roles and social roles [18]. Compared with men, women have more time to overlap work and family activities, so women face more conflicts between work and family roles [15, 19]. Due to the particularity of long-distance working, rural female migrant workers are unable to take into account family and work, and the conflicts they suffer are more obvious. Numerous studies have focused on the conflicts between family and work of career women and the influencing factors, but there is still a lack of the research on the conflicts between work and family of rural women. This study will make up for the deficiency of the research.

The research on subjective well-being has been mainly initiated in the United States since the 1950s and has entered the field of vision of Chinese researchers since the mid-1980s [20]. Researchers have believed that the influencing factors include neuroticism, extroversion, affinity, responsibility and other genetic factors [21] and social environment factors, such as age, gender, race, education, income, occupation, marital status, religious belief, etc. [22–24], as well as situation and daily experience factors and other external influencing factors (pleasant or adverse experience). Some studies have pointed out that genetic factors have a much greater impact, but it is difficult to change genetic factors, so studying external environment and situation factors is more important for improving people’s subjective well-being.

For the female group, once the conflicts between work and family cannot be effectively alleviated, it is easy to cause emotional exhaustion and reduce the efficiency of normal work and life [25]. The existing studies have generally argued that the conflicts between work and family would have a significant negative impact on subjective well-being, and work dissatisfaction would have a negative effect on life satisfaction [26]. The cross-cultural research on the relationship between the conflicts between work and family and the quality of life has found that the level of well-being perceived by Oriental women is closely related to family life [27]. However, Johada has considered that employment also has the potential to meet a variety of psychological needs of individuals, and if individuals leave the labor market, these psychological needs cannot be met, resulting in a negative influence on personal well-being; some studies have also believed that through work or family roles, individuals can obtain positive resources, so as to improve the functioning of the family system as a whole and enhance personal well-being [28]. Hence, the influence of work or family roles on subjective well-being is still controversial and needs to be further studied. Thus, in addition to the cognition of the conflicts between family and work, this paper considers the influence of work or family roles, so as to more systematically explore the influence mechanism of family and social roles on subjective well-being of rural women.

## Data and methods

### Study area profile and data source

Located in southwestern China, the Sichuan Province is upstream of the Yangtze River. It covers 486,052 km2 and has a population of 82.04 , with 42.92 million engaged in agricultural production [10]. The majority of the terrain is hilly (90%) and only 5.3% is flat plains. More than 60% of the region has an elevation of over 1000 m [3]. Meanwhile, the altitude exceeds 1000 m in more than 60% of the region. The annual net income of farmers in this province is 10,247 Yuan, which is 10.2% lower than the 2014 national average [10].

**Fig. 2:**
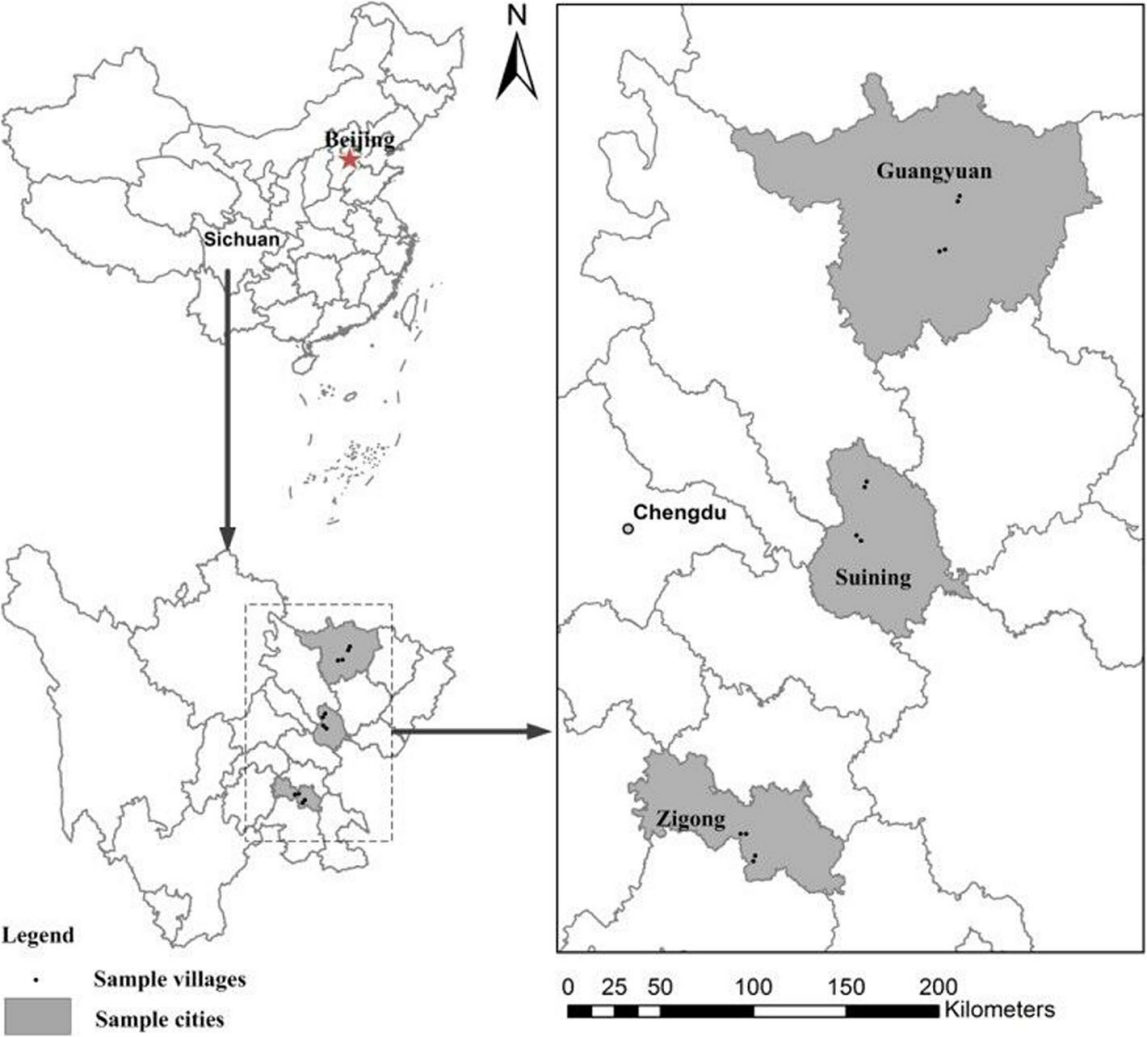
Locations of sample villages (Xu et al. 2017 [30])

The data used in this study are mainly from the rural household survey, which was implemented by the China Rural Development Survey Group in Sichuan Province in April 2016. The survey mainly focuses on the current situation and changes of rural labor force in Sichuan, involving many research topics such as role cognition, livelihood strategy, family production and so on. In order to ensure the representativeness of the sample, the sample covers 5 counties and 20 villages in Sichuan Province. In terms of sampling methods, this study used stratified sampling and equal probability random sampling [29], obtained 400 sample families and 415 female samples. After logical screening and elimination of incomplete information samples, a total of 380 valid samples were obtained. The maps of the spatial locations of the sample villages are shown in Fig. 2.

### Selection and definition of the model variable

In accordance with the previous research on subjective well-being and family-work conflict [21, 22, 31], well-being is a kind of subjective judgment, whose measurement is different from the objective gender equality indicator, and it adopts Likert scale, the classic method of measuring subjective attitudes in psychology. The judgment sentence of this paper is “do you feel happy in your life?” The respondents are asked to give the answers of “very unhappy - unhappy - general / unable to judge - happy - very happy” and give the indicator score of “1 - 5”. The higher score indicates that the respondents’ subjective well-being is higher, and their satisfaction with their current life is higher.

The conflicts and coordination between family and work also use Likert scale to set statements to be judged, to investigate the situation and cognition of the conflicts between family roles and career roles of rural women, the coordination and management of conflicts and the selections after facing conflicts (See Table 1). At the same time, drawing lessons from the setting of control variables in the studies from [30, 32] and other scholars, this paper divides the control factors affecting migrant workers’ employment location selections into family burden (including the number of elderly people who need to be supported in the family, the number of children in the family and the total population of the family), employment situation (including whether to go out work, employment industry, employment location and wage income) and personal characteristic (including educational level, marital status and age). The variable definition and data description involved in the model are detailed in Table 1.

**Table 1:** The design of the scale of the cognition of family-work role conflicts

Compositional Dimensions	Measurement Indicators	Statements to Be Judged	Judgment and Evaluation
*The cognition of the conflicts between family and work*	The situation and cognition of the conflicts between family and work	You often dispute with your spouse / families over work.	1 = Strongly Disagreeable 2 = Disagreeable 3 = General 4 = Agreeable 5 = Strongly Agreeable
	The situation and cognition of the conflicts between work and family	You often dispute with your spouse / families over taking care of the family.	
*The coordination of the conflicts between family and work*	The coordination and management of conflicts	If going out to work, you can well coordinate the conflicts between the family and the work.	
	The selections after facing conflicts	When there are conflicts between taking care of the family and going out to work, women should make certain sacrifices and take care of their families at home.	

Note: elderly individuals in the household were considered to be aged 65+ years; children in the household were classified as those aged 6 years and under

### Econometric model

Since “subjective well-being”, the dependent variable, is an orderly multinomial variable, in order to explore the influence of the conflicts between family and work (including cognition and coordination of conflicts, employment and family burden) on the subjective well-being of rural women, this study adopts the orderly multinomial logistic regression models to conduct the analysis.

In terms of model, weightage from the cluster and family is also considered, and the probability that the sample households are extracted as weightage. For example, there are 2000 households in village A, since each village draws 20 households, then for the sample households in the village, The probability of extraction is 20/2000.

## Result analyses

### Descriptive statistical analysis

Table 2 shows the results of descriptive statistical analysis. As shown in Table 2, rural women in the hilly areas interviewed had higher subjective well-being, 70% of them felt happy or very happy; in terms of conflict cognition, 58% of rural women did not clearly perceive WIF conflict, only 20% of women feel obvious WIF conflict, 59% of them have no obvious perception of FIW conflicts; when facing WIF conflict or FIW conflict, 65% of rural women have the confidence to coordinate the conflicts between the family and the work. It is worth noting that 72% of rural women in the hilly area agree or strongly agree that they should choose to take care of family, when conflicts arise.

**Table 2:** Model variables, definitions and data types

*Variable*	Definition and assignment	Min	Max	Mean	SD
*Explained variable*					
*SWB*	subjective well-being	1	5	3.74	0.82
*Explanatory variables*					
*cognition of family-work role conflicts*					
*WIF*	work-interfering-with-family conflict	1	5	2.49	1.00
*FIW*	family-interfering-with-work conflict	1	5	2.51	1.02
*CMC*	coordination and management of conflicts	1	5	3.47	0.98
*SFC*	selections after facing conflicts	1	5	3.80	1.15
*Control variable*					
*Family burden*					
*OLD*	old in the household	0	3	0.86	0.88
*CHI*	child in the household	0	3	0.37	0.59
*FAM*	Total number of the family	2	12	5.97	1.81
*Employment status*					
*WGO*	Whether go out to work	1	2	1.58	0.49
*PLA*	place of employment: 1 = in the county, 2 = in other counties of the province, 3 = in other provinces	1	3	1.37	0.73
*IND*	employment industry: 0 = unemployment/staying at home, 1 = farming, 2 = the secondary industry, 3 = the tertiary industryI, 4 = the tertiary industryII	0	4	1.69	1.39
*INC*	Yearly income of rural female labors	0	2.28	0.42	0.57
*Personal characteristics*					
*EDU*	Years of education of rural female labors	0	15	6.24	4.24
*MAR*	MAR = 1 for unmarried, MAR = 2 for married	1	2	1.90	0.31
*AGE*	Ages of rural women migrant laborers	18	64	44.94	13.03

Note: elderly individuals in the household were considered to be aged 65+ years; children in the household were classified as those aged 6 years and under

As far as the control variables, the average age of rural women in the hilly area is about 45 years old, and the age distribution is relatively average. About 90% of rural women in the hilly area are married, and the overall education level is low (The average value is only 6.21). About 85% of rural female labor force in the hilly area has only gone to junior high school and below. In the family characteristics, the population of rural families is relatively large, and the average household has nearly 6 people. More than half of the rural female laborers have one or more elderly people in their family, while nearly 70% of the households have no children under the age of 6; 42% of rural women in hilly regions choose to go out to work, and 38% of them are working at home, and nearly 80% of rural women choose to work in their hometown.

### Results

Table 3 shows the results of the effects of different conflict cognition and coordination on subjective well-being. Wherein, Model 1 is the regression results of the influences of the cognition and coordination of the conflicts between work and family on the subjective well-being of rural women, Model 2 adds a weight variable. Model 3 is the results after respectively adding characterizing the personal characteristic, family burden and employment situation of migrant workers and other control variables based on Model 2 in order to test the robustness of focus variables, Model 4 adds a weight variable. It can be seen from *Pseudo R*^*2*^ that the goodness of fit of Model 2 and Model 4 is higher than that of Model 1 and Model 2. This study also uses VIF test statistics to test whether there is serious multi-collinearity among independent variables of various models. The results show that all VIFs are less than 10, indicating that there is no serious multi-collinearity among independent variables.

**Table 3:** Ordered multi-classification logistic regression model

*Variables*	MODEL 1				MODEL 2				MODEL 3				MODEL 4			
	subjective well-being^a^ (OR)				subjective well-being^a^ (OR)				subjective well-beingb (OR)				subjective well-being^b^ (OR)			
	unhappy	general / unable to judge	happy	very happy	unhappy	general / unable to judge	happy	very happy	unhappy	general / unable to judge	happy	very happy	unhappy	general / unable to judge	happy	very happy
*WIF*	−0.61	−0.51	−0.81	−1.10^b^ (0.332)	−0.99	−0.76	−1.02	−1.31^a^ (0.270)	−0.79	−0.82	−1.07^a^ (0.345)	−1.35^b^ (0.259)	− 1.02	− 0.94	− 1.15^b^ (0.318)	− 1.45^b^ (0.236)
*FIW*	0.23	0.40	0.03	0.23	0.34	0.50	0.11	0.24	0.06	0.36	−0.07	0.10	0.03	0.37	−0.11	−0.01
*CMC*	0.28	0.64^a^ (1.897)	0.63^a^ (1.870)	0.60^a^ (1.822)	0.21	0.63^b^ (1.897)	0.62^b^ (1.852)	0.62^a^ (1.867)	0.47	0.74^a^ (2.09)	0.73^a^ (2.077)	0.66	0.58	0.89^b^ (2.443)	0.88^b^ (2.410)	0.83^a^ (2.291)
*SFC*	−1.27^a^ (0.280)	−1.32^b^(0.267)	− 1.21 (0.298)	−0.99	− 1.41^b^ (0.244)	− 1.51^b^ (0.220)	−1.42^**b**^ (0.242)	− 1.15^a^ (0.315)	−1.58^a^ (0.207)	− 1.54^a^ (0.215)	−1.42^a^ (0.242)	− 1.16	−1.85^a^ (0.158)	− 1.79^a^ (0.166)	−1.68^a^ (0.187)	− 1.36
*OLD*									−1.51^a^ (0.221)	−0.93	−1.05	− 0.91	−2.16^c^ (0.115)	−1.17^c^ (0.311)	− 1.29^c^ (0.276)	−1.18^c^ (0.309)
*CHI*									−2.46^b^ (0.085)	−2.67^b^ (0.069)	−2.64^b^ (0.072)	−1.80	− 2.71^c^ (0.066)	− 2.75^c^ (0.064)	− 2.83^c^ (0.059)	−1.88^c^ (0.153)
*FAM*									0.75	0.87^a^ (2.382)	0.89^a^ (2.44)	0.70	0.86^c^	0.86^c^ (2.363)	0.89^c^ (2.457)	0.69^b^ (1.952)
*WGO*									−2.00	−2.46	−1.91	−2.69	− 2.88	− 2.60	−2.19	−3.64^a^ (0.026)
*PLA*									−0.77	−0.61	−0.65	−0.23	−1.40	−0.87	−1.08	− 0.59
*IND*									−1.30	−1.07	−1.05	− 1.21	−1.48	− 1.21	−1.17	− 1.51
*INC*									−1.38	−1.15	−1.35	− 1.87	−1.24	−0.53	−0.84	− 1.65
*EDU*									0.26	0.28	0.33^b^ (1.395)	0.41^b^ (1.502)	0.35^b^ (1.413)	0.36^b^ (1.432)	0.41^c^ (1.505)	0.47^c^ (1.593)
*MAR*									0.71	2.05^a^ (7.765)	2.32^a^ (10.15)	1.57	0.70	2.25^a^ (9.450)	2.44^b^ (11.439)	1.84
*AGE*									−0.03	−0.05	−0.06	−0.03	0.02	0.00	−0.02	0.01
*N*	380				380				380				380			
*Likelihood ratio tests*	Chi-Square = 47.25^c^ df = 16)				Chi-Square = 44.04^c^ (df = 16)				Chi-Square = 99.47^c^ (df = 56)				Chi-Square = 106.1^c^ (df = 56)			
*Pseudo R*^*2*^	0.057				0.062				0.12				0.132			

Note: Model 2 and Model 4 have added weights. Robust standard errors are in the parentheses. ^a^, ^b^ and ^c^ represent statistical significance at 0.1, 0.05 and 0.01, respectively. ^a,b^ The reference group is ones who chose very unhappy

From the chi-square test statistics of Models, it is seen that all models have passed the overall significance test, indicating that in each model; at least one independent variable is significantly correlated with the dependent variable. At the same time, the explanatory ratio of the independent variable to the dependent variable variance of the model is between 5.7 and 12%. Through the comparison and analysis of Model 2 and Model 4, it is found that after adding family burden and employment situation, the results of the effect on subjective well-being are relatively stable. Therefore, the subsequent result analysis is mainly based on the results of Model 4.

As shown in Table 3, the cognition of work interference family conflict has a marked negative impact on subjective well-being. Compared with rural women who feel very unhappy, with every one unit increase in the conflicts between work and family, the probabilities of feeling happy and very happy decrease by 68.2 and 76.4%, respectively, which is basically consistent with the results of previous studies (the conflicts between work and family would have a negative influence on subjective well-being); however, family interference work conflict has no significant effect on subjective well-being;

In the aspect of the coordination of conflicts, the coordination ability of subjective cognition has a noticeable positive influence on subjective well-being. With every one unit increase in subjective coordination ability, the probabilities of feeling general, happy and very happy increase by 144.3, 141 and 129.1%, respectively. The evaluation of self-coordination ability is a part of self-efficacy. Women with high self-efficacy face up to their own abilities to deal with various stresses in life from a confident perspective, and they will be more active in life, so well-being will be enhanced as well.

The balance of conflicts has an obvious negative effect on subjective well-being, which is reflected in the fact that compared with rural women who feel very unhappy, with every one unit increase in the willingness of rural women to choose to stay at home, the probabilities of feeling unhappy, general and happy decrease by 84.2, 83.4 and 81.3%, respectively.

In control variables, family burden has a remarkable impact on the subjective well-being of rural women as well, which is reflected in the fact that the number of children has a significant influence on the subjective well-being of rural women, and compared with rural women who feel very unhappy, with every one unit increase in the number of children in the family, the probabilities of feeling unhappy, general, happy and very happy for rural women decrease by 93.4, 93.6, 94.1 and 84.7%, respectively; with every one unit increase in the number of the elderly man in the family, the probabilities of feeling unhappy, general, happy and very happy for rural women decrease by 88.5, 68.9, 72.4 and 69.1%, respectively, which shows that the impact of family burden on women’s subjective well-being is negative. However, compared with rural women who feel very unhappy, with every one unit increase in the total population of the family, the probabilities of feeling general, happy and very happy for rural women increase by 136.3, 145.7 and 95.2%, respectively, this is consistent with Frank’s conclusions that family size has a positive impact on women’s subjective well-being [22]. The survey from The Institute of Population and Labor Economics, Chinese Academy of Social Sciences (IPLE-CASS) has also found that family size and family structure have an important effect on family well-being, while single-person families, single-parent families, individual divorce, widowhood and absence of father or mother lead to the lack of the proper support and care in the individual’s life, and family affection, love, etc. would have a certain negative impact on the individual’s psychology, so as to reduce the well-being of individuals and families.

Educational level has a more significant positive impact, and marital status has a remarkable influence as well, which is reflected in the fact that married women’s subjective well-being is higher than unmarried women’s. At the same time, it can be seen that the working factors, which include whether to go out to work, employment location, employment industry and income, have no obvious influence on the subjective well-being of rural women.

## Discussions, conclusions and implications

### Discussions

Based on the large-scale rural household survey data from Sichuan Province, China, this study evaluates the influence of the cognition and coordination of conflicts between family and work on women’s subjective well-being from the perspective of rural female individuals. Compared with the existing research, the marginal contributions of this study are as follows: firstly, this study focuses on rural women, which are a group rarely concerned by previous studies; secondly, based on the Western conflict scales, this study constructs the conflict scale suitable for rural women in China, adding family burden, employment situation and other factors, so as to better reveal the influence of the conflicts and coordination between family and work on subjective well-being of rural women in China, which can help us more conveniently provide the specific guidance for the improvement of life satisfaction of rural women.

It is seen from this study that all the three factors of the conflicts between work and family have an impact on well-being, which verifies the research results of Bai et al. and Noraini that the conflicts between work and family have a negative influence on well-being [5, 31]. However, this study has also found that the most relevant to well-being is work interference family conflict, followed by the balance between work and family and the confidence in the coordination of conflicts, while family interference work conflict is not correlated with well-being. The results also verify the results of the cross-cultural research from Samuel et al., Kossek and Ozeki on the relationship between the conflicts between work and family and the quality of life [27, 33]. They have argued that many relations in the work-family structure are similar in Eastern and Western cultures, but their natures and influences on quality of life are different. Control variables have also shown that for rural women, family structure and family size have a marked effect on life well-being, while they present no sensitivity to working factors. This indicates that the level of well-being perceived by women, especially rural women, is closely related to family life. In the cognition of rural women, most of them agree with the traditional gender division of labor. With strong awareness of “homemaking women”, they are obviously bound by tradition, and their positioning of women and their roles tend to be traditional. At the same time, rural women would stabilize their own mentality in balancing their family and work and be more satisfied with their life. Thus, their subjective evaluation of life is generally high.

In addition, in this study, there are some deficiencies, which can be further dealt with in the future research. For example, this study has found that family roles and social roles have different effects on women’s subjective well-being, and the future research can further quantitatively explore the correlation between self-positioning social roles and well-being. Compared with developed countries, China’s current urbanization rate still has much room for improvement. With the development of economy, more and more female laborers will be transferred from the agricultural sector. In this case, the panel data are needed to better reveal the dynamic changing process of the conflicts and balance between families and work on the quality of life and subjective well-being, whereas this study is only based on the static cross-section data, which can be further dealt with in the future research.

### Conclusions and implications

Through the representative survey of 380 rural women of Sichuan Province and the analysis on the influence of role cognition and employment strategy selections of rural female laborers, the following main conclusions are drawn:

1. The level of subjective well-being of rural women is generally high, and 70% of women feel happy or very happy. 58% of rural women have no obvious perception of work interference family conflict, while 59% of rural women have no obvious perception of family interference work conflict, and 20% of rural women have obvious perception of work-family conflict, indicating that the surveyed rural women have higher subjective evaluation of life.
2. The most relevant to subjective well-being of rural women is work interference family conflict, and with every one unit increase in the conflicts between work and family, the probabilities of feeling happy and very happy decrease by 65.5 and 74.1%, respectively. The more relevant to subjective well-being of rural women is the balance between work and family and the confidence in the coordination of conflicts, and with every one unit increase in the willingness of rural women to choose to stay at home, the probabilities of feeling unhappy, general and happy decrease by 79.3, 79.5 and 75.8%, respectively, whereas with every one unit increase in the confidence in the coordination of conflicts, the probabilities of feeling general and happy increase by 109 and 107.7%, respectively.
3. Facing the conflicts between family and work, 72% of the rural women agree or strongly agree that when there are conflicts between taking care of the family and going out to work, they should choose to take care of their families at home. The improvement of their life satisfaction is significantly related to their self-positioning social roles.

In order to alleviate the conflicts between family and work of rural women and enhance the subjective well-being of women in rural areas, there is a need to improve women’s educational level, form clear role expectations and establish role compensation consciousness. Moreover, it is necessary for women to fundamentally establish the key responsibility according to their own abilities and objectively evaluate the pressures in the reality. In this way, they can truly better deal with inner contradictions and psychological conflicts.

## Data Availability

The data that support the findings of this study are available from China Rural Development Survey Team, but restrictions apply to the availability of these data, which were used under license for the current study, and so are not publicly available. Data are however available from the authors upon reasonable request and with permission of China Rural Development Survey Team. The data will not be shared because this is the confidential data of the China Rural Development Survey Team.

## Abbreviations

FIW: Conflict and Family-interfering-with-work
IPLE-CASS: The Institute of Population and Labor Economics, Chinese Academy of Social Sciences
VIF: Variance Inflation Factor
WIF: Work-interfering-with-family
